# Evaluating recruitment, retention and adherence patterns in the GET FIT fall prevention exercise trial in older, postmenopausal cancer survivors

**DOI:** 10.21203/rs.3.rs-5968659/v1

**Published:** 2025-02-10

**Authors:** Jessica Sitemba, Mary Crisafio, Fuzhong Li, Elizabeth Eckstrom, Kerri M. Winters-Stone

**Affiliations:** Oregon Health & Science University; Oregon Health & Science University; Oregon Research Institute; Oregon Health & Science University; Oregon Health & Science University

**Keywords:** exercise training, feasibility, older adult, cancer survivorship, fall prevention

## Abstract

**Purpose:**

The GET FIT trial tested fall prevention exercise approaches in older (50–75 years) post-chemotherapy, postmenopausal cancer survivors. We describe recruitment, retention, and adherence patterns from GET FIT to inform future trials.

**Methods:**

Participants were recruited through multiple strategies (e.g., cancer and research registries, clinician referral, outreach, electronic health record (EHR) screening) and were randomized to one of three supervised, facility-based, group exercise programs for six months. We compared effectiveness of accrual across recruitment strategies, examined characteristics of women who completed the interventions to those who withdrew, and women with good (≥ 50%) versus poor (< 50%) adherence to training.

**Results:**

Of 1490 interested women, 442 women were eligible, randomized, and received the assigned intervention (30% accrual rate). Accrual was similar across recruitment strategies, except for EHR screening which yielded no accruals. Retention over 12 months was 87% with most dropouts occurring within the first month. There were no differences in baseline characteristics between women who did or did not drop out. Poor adherers (n = 60) had higher baseline BMI, comorbidities, pain, disability and lower physical functioning (p < 0.05) compared to more adherent women (n = 377).

**Conclusions:**

A variety of recruitment strategies appear to be effective for enrolling older, postmenopausal cancer survivors into a facility-based exercise trial, except for directly approaching women identified through the EHR. Women with poorer health were at risk for study drop-out and poor adherence to exercise.

## INTRODUCTION

Cancer and its treatment can exacerbate age-related declines in physical functioning and increase fall risk [1–3]. The GET FIT trial was a 3-arm randomized controlled trial testing the efficacy of strength training or tai ji quan training compared to a placebo exercise (stretching) condition on fall rates in postmenopausal women who received chemotherapy for cancer [4]. The exercise interventions in GET FIT were supervised, facility-based, group exercise programs to better ensure fidelity and safety of exercise programs, optimize outcomes with supervised exercise, utilize social support to promote retention and adherence, and economically deliver a training program. Enrolling cancer survivors into supervised, facility-based group exercise programs and achieving high adherence and retention rates is a challenge for clinical exercise research, particularly with a need for adequately powered sample sizes to detect changes in event rates such as falls [5]. Facility-based group exercise interventions can impose additional constraints over non-interventional or even therapeutic trials, including travel to a facility, substantial time commitment to attend programs, and scheduling constraints with set class days and times that can be barriers to exercise [6]. While women cancer survivors express interest in engaging in exercise programs [7], a woman’s readiness to exercise after cancer can be impacted by her starting fitness level, persistent treatment-related symptoms (e.g., pain, fatigue, and neuropathy) and lack of self-efficacy as well as other social and environmental factors (e.g., barriers to facilities and lack of social support), potentially contributing to poor adherence or drop out [8, 9]. Older cancer survivors may be less likely to express interest in exercise interventions than their younger counterparts over concerns about their safety, appropriateness of exercise, and study burden [10].

While randomized controlled trials of post-treatment exercise interventions in postmenopausal women cancer survivors describe recruitment approaches and report study retention and exercise adherence [11–15], there have been no detailed reports comparing success of different recruitment strategies or characterizing study retention and adherence patterns that could inform the design and execution of future trials. Given the need for rigorous controlled trials that test exercise efficacy on outcomes important to older cancer survivors (e.g., falls, fractures, frailty, disability), further detailed reporting on trial accrual, retention, and adherence from completed clinical exercise trials can benefit the research community and help guide implementation into practice. Thus, the aims of this secondary analysis of data from the GET FIT trial [4] are to: 1) describe and compare accrual rates across a variety of recruitment strategies, 2) describe study retention and adherence and and compare characteristics of study completers versus non-completers and of good versus poor adherers to exercise programs.

## METHODS

### Study Design and Procedures

Details outlining the study setting, design, participants, procedures, and interventions have been previously published [4]. Briefly, GET FIT was a 3-arm randomized controlled trial of postmenopausal cancer survivors aged 50–75 years who had completed chemotherapy. The sample size powered to test for intervention differences in fall rates was n=456 (accounting for planned attrition of 20%), or 152 per study arm. Enrolled participants were randomized into one of three supervised, facility-based, group exercise interventions (strength training, tai ji quan, or stretching [control]) twice-weekly for 6 months. After supervised training stopped, women were provided an exercise video and written guide to follow at home on their own and were reassessed 6 months later. Participants underwent testing sessions at baseline, 3, 6 and 12 months which included completing questionnaires and physical performance tests previously described [4]. Questionnaire-based measures included in this analysis were baseline demographic and clinical characteristics, comorbidities (Functional Comorbidity Index and Charlson Comorbidity Index) [16], fear of falling (Survey of Activities and Fear of Falling in the Elderly-SAFFE) [17], and lifestyle physical activities outside of the exercise intervention (CHAMPS Physical Activity Questionnaire for Older Adults) [19]. Falls were tracked and collected via monthly surveys. The exercise classes were held at Oregon Health and Science University (OHSU), an academic medical center, plus six community locations in and around the Portland metro area in Oregon. Community settings were strategically selected to reach different geographical sectors of the Portland metro area to reduce barriers to participation related to time and travel. Classes were held on specific days and times, lasted 60 minutes per session, were led by a certified group exercise instructor, in groups of 15–20 participants per class. All study procedures and written informed consent, were reviewed, and approved by the OHSU Institutional Review Board (IRB #8560). The trial is registered with ClinicalTrials.gov (NCT01635413).

### Study Sample

Inclusion criteria were: 1) diagnosed with stage I-III cancer other than cancer of the brain or spinal cord, 2) completed chemotherapy >3 months prior to enrollment, 3) aged 50–75 on date of enrollment, 4) physically underactive (<60 minutes of moderate intensity exercise per week the month prior to enrollment), 5) ability to provide informed consent, and 6) free of contraindications to moderate-intensity exercise.

### Recruitment

Five different strategies were used to recruit women into the trial. These included (a) cancer registries, (b) clinician referral, (c) mailed invitations to past research participants, (d) community outreach (e.g., attending cancer and health related events, posting flyers, email blasts), and (e) screening and outreach through electronic health records (EHR). Accrual rates for each strategy were calculated as the number of women who were randomized and received the intervention (n=442) out of the number of interested and eligible women within each recruitment strategy. We briefly describe each strategy in detail below.

#### Cancer Registries

We utilized two cancer registries to recruit participants, the Oregon State Cancer Registry (OSCaR) and the OHSU Hospital Cancer Registry. OSCaR is a population-based tumor registry run by the Oregon Health Authority that collects and analyzes information on cancer cases in Oregon. To partner with the registry, the GET FIT protocol and recruitment approach was approved by the OSCaR advisory board and Oregon Health Authority IRB. The OSCAR registry identified potential participants by age, cancer site, diagnosis date and treatment type. Each woman’s physician of record was sent a letter from OSCaR informing them that their patient would be sent a letter about the trial. In accordance with HIPAA5145 regulations, the OSCaR registry would next mail women a joint OHSU-OSCaR information letter describing the study along with a pre-paid response form to allow the study team permission to contact them about the study (n=5145 letters sent). In addition to OSCaR, the OHSU registry was utilized to identify more potentially eligible women who were treated for cancer at OHSU but had not received a letter through OSCaR (n=824 letters sent).

#### Research Repository

The research team has an IRB-approved research repository of persons who provided permission to be contacted for future study opportunities from prior recruitment efforts. Women who met basic eligibility criteria (e.g., time since diagnosis, treatment type) and lived in the Portland metro area were sent an information letter (n=573 letters sent) and were asked to contact study staff by email or phone if they were interested in learning more about the trial. Women in the repository were not directly called by the study team.

#### Community Outreach

Recruitment flyers were placed in OHSU clinics and in local hospitals, clinics, and community centers. Direct outreach, via engagement of several community cancer-based organizations (e.g., formal and informal support groups, charities), was used to distribute information about the study through email listservs, newsletters and events. General study advertisements were also posted on social and print media, including small community newspapers.

#### Clinician Referral

OHSU oncologists were encouraged to refer potentially eligible patients to the study. If the clinician received permission from the patient for the study team to contact her, s/he then provided study staff with the patient’s medical research number (MRN) that included the patient’s contact information.

#### EHR Screening

OHSU clinicians provided study staff permission to review the EHR of their patients to directly identify potentially eligible women. Once a woman was identified, study staff called them on the phone to discuss the study. This strategy was considered unique from the other recruitment strategies since these women were approached about the study before they received information about it from their provider or elsewhere and/or expressed interest in response to a recruitment effort.

### Retention and Adherence

Multiple strategies were used to promote retention of participants during the study period including the following: 1) assignment of every woman to a supervised, group exercise program, including controls, 2) free close-by parking for study visits and exercise classes, 3) a $10 gift card after completion of each testing appointment, 4) small study tokens like a study magnet and t-shirt, and 5) birthday cards mailed to participants. Multiple strategies were used to promote adherence to exercise classes including: 1) professional exercise instruction to provide coaching, individual tailoring, and motivation, 2) one week of exercise class “orientation” to provide education about exercise and establish rapport, trust and support prior to starting exercise classes, 3) regular communication between instructors and participants during and outside of classes about exercise tolerance and missed sessions, 4) fostering group dynamics within exercise classes to engender social support from peers, and 5) in-class celebrations to mark the midpoint and end of the exercise program.

Study retention was calculated as number of completers out of the number of women who began the study interventions. Completers were defined as women who finished the 6-month exercise program and the 12-month study follow-up visit. Adherence was calculated as the proportion of attended classes out of those prescribed. Since women were prescribed twice weekly exercise classes, we created a cutoff of 50%, or an average of 1 or more classes per week, where ≥50% was deemed good adherence and < 50% deemed poor adherence.

### Statistical Analysis

Descriptive analyses were run to calculate mean, standard deviation and/or proportions as appropriate for each outcome. A chi-square analysis was conducted to compare differences in accrual rates across recruitment strategies. Differences in baseline demographics, clinical characteristics and study outcomes between completers and non-completers and between good and poor adherers were examined by unpaired *t* tests for continuous variables and chi-square or Fisher’s exact test for categorical variables. All statistical procedures were performed using the SPSS statistical software program, version 24.0 (SPSS, Inc., Chicago, IL, USA); a 2-tailed p-value <0.05 was considered statistically significant.

## RESULTS

### Study Sample

**Figure 1:**
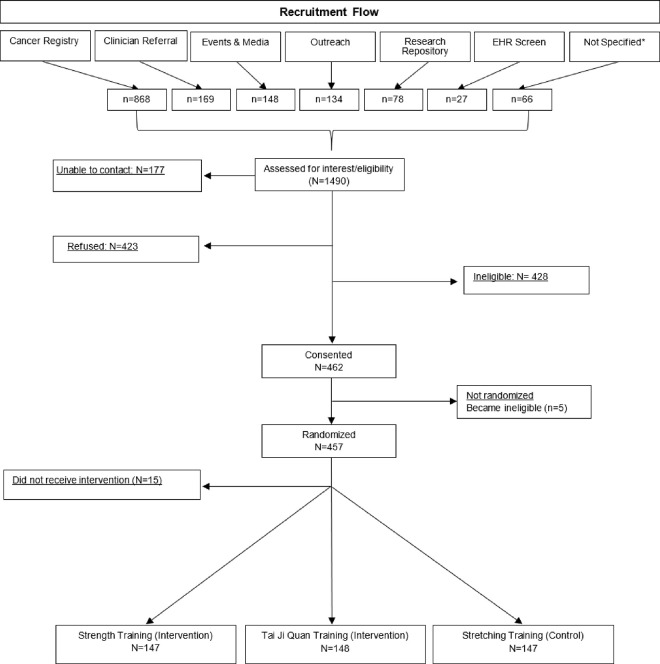
Recruitment Flow by Strategy through Randomization *Not specified: women who expressed interest by contacting study staff who were unable to get a successful follow-up contact to find out how they heard about the study

Participants were enrolled into the GET FIT study between January 2013 and September 2015. A total of 1490 women, identified through our various recruitment strategies, were screened per study eligibility (Fig 1). Of these, 457 were eligible and randomized. Fifteen women either became ineligible (i.e., cancer recurrence) or discontinued study participation prior to the start of the interventions (Fig 1), resulting in an overall accrual rate of 30% over a 33-month recruitment period. The accrual rate of interested persons was similar across all initial strategies, ranging from 29%–37%, except for EHR screening which resulted in zero accruals. The cancer registries were the most fruitful (n=868), and with a similar accrual rate as other approaches, yielded the highest number of enrolled participants (n=279; 32%) (Table 1). Recruitment from the research repository had a smaller yield (n=78), but the highest accrual rate (37%)

### Retention

**Figure 2:**
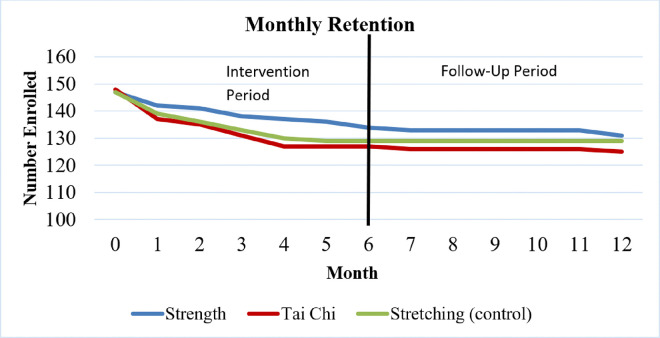
Retention of GET FIT participants by study arm during the supervised exercise intervention and follow-up periods.

Of the 442 women who began the study interventions, 385 (87%) completed both the 6-month exercise program and the 12-month follow-up testing visit. Of the 57 (13%) non-completers, 52 did not complete the 6-month exercise program and an additional 5 did not complete the final testing visit, resulting in retention rates of 88% and 87% during the first and second halves of the study, respectively (Figure 2). The majority of non-completers (n=24) withdrew within the first month of the study (Figure 2). Reasons for withdrawal among the non-completers included being too busy (n=18), disliking the class assignment (n=9), no longer interested (n=8), family emergency (n=5), poor health (n=4), schedule conflicts (n=4), transportation issues (n=3), musculoskeletal issues (n=3), and deceased (n=3). There were no significant differences in the number of non-completers across the study arms (p=.471). The only significant difference in baseline characteristics between non-completers and completers was the SAFFE score, where non-completers reported more worry about falling when performing activities of daily living at baseline compared to the completers (p=0.03) (Table 2).

### Adherence

**Figure 3:**
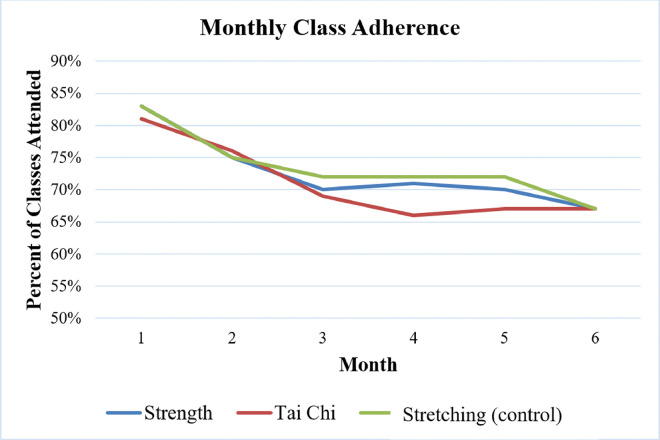
Adherence to GET FIT interventions (% of prescribed sessions attended) by study arm over six months.

Adherence over the 6-month active exercise intervention averaged 73%, 71%, and 74% for the strength, tai ji quan, and stretching (control) groups, respectively, with no significant difference across study groups. Average adherence rates to supervised classes were highest in the first month of the program (82%), then showed a steady decline (by about 10%) for the next two months of the program and remained steady after that (Figure 3). Women who were considered poor adherers had significantly higher BMI (p<0.01), more comorbidities (p<0.01), higher self-reported pain (p<0.05), lower self-reported physical function (p<0.05), greater disability (p<0.05), and were more likely to be single (p=0.01) compared to good adherers (Table 3).

## DISCUSSION

Overall, we were able to enroll and retain a large number of older, postmenopausal cancer survivors into a large clinical trial of 6-month supervised, facility-based group exercise interventions testing the efficacy of exercise-based fall prevention approaches. We used five different strategies to recruit women, reaching our target enrollment (n = 456) with a 30% accrual rate over a 33-month period. This accrual rate was similar to or higher than that reported in several other randomized controlled trials of cancer survivors that included a supervised, facility-based, group exercise intervention lasting 6 months or more [9, 15, 20, 21]. Hayes et al reported a 63% accrual rate to an 8-month long facility-based group exercise intervention in post-surgical breast cancer patients by provider referral at the time of surgery [12]. Similar to other exercise trials, our use of cancer registries was the most effective recruitment tool for enrolling a large sample [9, 15, 20, 21]. While the relative accrual rate from the registries was comparable to other methods, the number of women who can be reached through the registries is much higher than other approaches, yielding higher absolute numbers of enrolled participants. State and hospital cancer registries may be an effective resource for enrolling large numbers of participants into trials, but research teams should also consider the administrative challenges that this recruitment method may pose including fees, additional IRB approvals, and coordination of large mailings and/or phone calls, all of which can make this high yield approach resource intensive. In comparison, directly calling patients identified through the EHR was both time-intensive and unfruitful. While providers gave the research team permission to contact their patients, they did not discuss the study with their patient beforehand nor did the patient receive information about the study prior to the call from the research team, likely leaving the patient unready to receive a recruitment call. In an improved approach, we now encourage the provider to send an electronic message to their patient introducing and endorsing the study, prior to any contact from the study team. This approach, though being more fruitful, will only work for patients who opt into patient portals, such as MyChart. For patients who do not opt in, sending information by mail or providing it at a clinic visit could also be a “soft introduction” to the study and lead to a more successful recruitment call.

Both retention and adherence rates in our trial were similar to or higher than rates reported in other randomized controlled trials of female cancer survivors that included a supervised facility-based group intervention lasting 6 months or more [12, 13, 15, 22]. Overall, there were no differences in baseline characteristics between completers and non-completers with one exception. Non-completers reported significantly higher worry about falls compared to those who finished the study. Since our trial was focused on fall prevention, this is a curious paradox, but might be explained in part by the known relationship between fear of falling and self-restricted activity levels [23]. Women in our study who had a greater fear of falling, and who were also underactive per study eligibility criteria, may have felt more worry about falls as they started a new exercise program, though this concern was not specifically cited by any of these women as a reason for withdrawing. Since activity restriction can lead to a greater risk of falls and since exercise offers protection against falls, including specific strategies to reduce concern for falling, such as cognitive behavioral therapy, should be considered.

The benefits from exercise are often stronger with greater adherence to exercise [24–26]. In our study a small proportion of participants (14%) attended less than half of the prescribed classes and understanding this subgroup could inform adherence strategies in future studies. Participants with poor attendance had higher BMI, more comorbidities, self-reported pain, and disability, lower self-reported physical function and were more likely to be single. Other studies have also shown high BMI as a predictor for low adherence to exercise [27, 28]. One study of breast cancer survivors exercising during treatment reported that less physically fit women were more likely to have poor adherence to exercise prescriptions [29], while others have reported that low self-efficacy and greater physical limitations are key determinants of poor exercise adherence after cancer treatment [31, 32]. Since survivors with poorer health are at greatest risk of further physical decline and therefore could benefit from supervised exercise, incorporating strategies to improve adherence in this subgroup is important. Despite having a second instructor in class to modify programs for participants with low initial functioning (e.g., wall pushups instead of knee pushups), this approach was less successful in retaining these participants. Perhaps the need to have modified exercises within a group setting may have led to feelings of low self-confidence or efficacy which are known to lead to poor adherence. Confidence-enhancing strategies may be needed, especially during the initial acclimation period, for women with low baseline functioning and movement efficacy to slowly ease into structured, prescribed exercise programs.

There are limitations to interpreting our findings. Our sample was well educated and mostly identified as non-Hispanic white living in a metropolitan area. Thus, it is possible that our findings may not translate directly to a more racially and ethnically diverse patient population living in different geographic regions. Future studies should increase participant diversity and thus the generalizability of the results by using recruitment methods like community-based participatory research and physician-informed and engaged referrals, since engagement in clinical trials research among underserved groups is notably low. Additionally, participant age was limited to women who were no older than 75 years of age to better test the efficacy of the interventions on falls associated with chemotherapy rather than advanced age and/or age-related comorbidities. Barriers to engagement in clinical research, exercise tolerance and adherence may differ among women over 75 years of age. Importantly, this group is also underrepresented in exercise oncology research and deserves more attention as the proportion of cancer survivors over the age of 75 will double in the coming decades.

The GET FIT study demonstrated success in recruiting older postmenopausal cancer survivors into a large exercise clinical trial using strategies primarily involving state and hospital cancer registries. Once enrolled women were highly likely to complete participation in 6 months of supervised, facility-based exercise, and study follow-up visits over a year. However, women’s initial health status and physical function affected our ability to train and retain this small, yet important, subgroup of women who may have benefitted from structured exercise. Future exercise oncology studies need to consider targeted strategies to improve retention and adherence among women with poorer health throughout intervention and follow up periods.

## Figures and Tables

**Table 1.** Recruitment strategies and accrual rates in the GET FIT trial

	Cancer Registry	Clinician Referral	Community outreach^b^	Research Repository	EHR^c^ Screening	Not Specified	Total
Number assessed for eligibility	868	169	282	78	27	66	1490
Received intervention	279	50	80	29	0	4	442
Accrual Rate^a^	32%	30%	28%	37%	0%*	6%	30%

a Accrual rate is number randomized and received the intervention (n=442) divided by number of interested and eligible women within each recruitment strategy

b combines Events & Media and Outreach

c Electronic Health Record

* Significantly lower enrollment rate than other recruitment strategies p<0.01

**Table 2:** Baseline characteristics of completers and non-completers in the GET FIT trial. Data presented as mean (M), standard deviation (SD), and range for continuous data or % of sample for categorical data.

	Completers	Non-Completers	
	(n = 385)	(n = 57)	
Characteristic	M (SD)	M (SD)	p-value
Group Assignment			
Strength	34%	28%	0.47
Tai Ji Quan	33%	40%	
Stretching	34%	32%	
Age (years)	62.0 (6.4)	62.3 (5.8)	0.75
Cancer Type			
Breast	71%	74%	0.25*
Cervical	1%	0%	
Colon	7%	4%	
Liver	0%	0%	
Lung	2%	5%	
Lymphoma	4%	4%	
Ovarian	6%	2%	
Pancreatic	0%	2%	
Urinary/Bladder	0%	0%	
Uterine	3%	4%	
Other	6%	7%	
Cancer Stage†			
I	28%	21%	0.72
II	40%	40%	
III	26%	25%	
Race			
White	90%	88%	0.30**
African American/Black	2%	5%	
Native Hawaiian/Pacific Islander	1%	0.0%	
Native American/Alaskan Native	1%	2%	
Asian	3%	2%	
Ethnicity			
Hispanic	2%	2%	0.62
Non-Hispanic	96%	97%	
Marital Status			
Married/Partnered	58%	56%	0.82
Education			
High School/GED	21%	33%	0.11
> High School	79%	66%	
Employment			
Retired	42%	53%	0.14
Full time	29%	14%	
Part time	17%	21%	
Homemaker	4%	2%	
Unemployed	8%	11%	
Comorbidities			
Charlson Comorbidity Index	1.9 (1.5)	2.2 (2.0)	0.25
Functional Comorbidity Index	1.9 (1.6)	2.3 (2.1)	0.12
BMI (kg/m^2^)	29.5 (6.6)	30.4 (7.2)	0.37
Fall History (last 6 months)			
>=1 Fall	21%	26%	0.20
Injurious Fall (out of fallers)	53%	60%	0.42
Pain Severity	1.3 (1.5)	1.6 (1.6)	0.22
Pain Interference	1.0 (1.6)	1.4 (2.1)	0.17
Neuropathy (yes)	42%	32%	0.50
SAFFE	0.24 (0.35)	0.40 (0.52)	0.03
LLFDI disability limitation	77.3 (14.7)	76.5 (16.8)	0.70
LLFDI overall function	68.2 (11.0)	66.6 (12.1)	0.33
LLFDI function lower extremity	81.1 (14.4)	79.5 (15.6)	0.43
LLFDI function-advanced lower extremity	63.3 (16.3)	61.2 (16.9)	0.36

† Numbers may not add up to 100 due to missing data

* Significance test compares breast vs other

** Significance test compares White vs other

**Table 3:** Baseline characteristics of participants who attended ≥ or < 50% of prescribed exercise classes. Data presented as mean (M), standard deviation (SD) and range for continuous data or *%* of sample for categorical data.

	Adherence ≥ 50%	Adherence < 50%	
	(n = 377)	(n = 60)	
Characteristic	M (SD)	M (SD)	p-value
Age (years)	62.1 (6.3)	61.9 (6.5)	0.86
Cancer Type			
Breast	71%	72%	0.93*
Cervical	1%	3%	
Colon	7%	3%	
Liver	0%	0%	
Lung	3%	2%	
Lymphoma	4%	3%	
Ovarian	6%	3%	
Pancreatic	0%	0%	
Urinary/Bladder	0%	0%	
Uterine	3%	5%	
Other	5%	8%	
Cancer Stage†			
Donť know stage	5%	8%	0.76
I	28%	22%	
II	40%	43%	
III	27%	25%	
No Stage	1%	2%	
Race			
White	91%	83%	0.03**
African American/Black	2%	5%	
Native Hawaiian/Pacific Islander	0%	3%	
Native American/Alaskan Native	1%	0%	
Asian	3%	2%	
Ethnicity			
Hispanic	1%	2%	0.60
Non-Hispanic	96%	98%	
Marital Status			
Married/Partnered	60%	43%	0.01
Education			
High School/GED	21%	33%	0.29
> High School	79%	66%	
Employment			
Retired	43%	43%	0.32
Full time	28%	20%	
Part time	17%	22%	
Homemaker	5%	2%	
Unemployed	8%	13%	
Group Assignment			
Strength	33%	32%	0.96
Tai Ji Quan	34%	35%	
Stretching Control	33%	33%	
Comorbidities			
Charlson Comorbidity Index	1.9 (1.5)	2.5 (1.7)	0.002
BMI (kg/m^2^)	29.2 (6.5)	32.2 (7.2)	0.001
Fall History (last 6 months)			
≥1 Fall	21%	22%	0.57
Injurious Fall (out of fallers)	51%	77%	0.08
Pain Severity	1.3 (1.5)	1.9 (1.8)	0.03
Pain Interference	1.0 (1.6)	1.5 (2.0)	0.06
Neuropathy (yes)	43%	30%	0.15
SAFFE	0.25 (0.37)	0.34 (0.47)	0.15
LLFDI disability limitation	77.7 (14.8)	0.02	0.02
LLFDI overall function	68.5 (10.9)	0.02	0.02
LLFDI function lower extremity	81.6 (14.4)	<0.01	<0.01
LLFDI function-advanced lower extremity	63.7 (16.2)	0.02	0.02

† Numbers may not add up to 100 due to missing data

* Significance test compares breast vs other

*** Significance test compares Caucasian/white vs other

## Implications for Cancer Survivors:

Women with poorer initial health may need additional retention strategies to help them stick with supervised, facility-based, group exercise.
